# Wide Margins are Still Relevant: A Case of a Well-Circumscribed Borderline Phyllodes Tumor With a Satellite Nodule in a Re-excision Margin

**DOI:** 10.7759/cureus.79890

**Published:** 2025-03-01

**Authors:** Ellery H Reason, Gina Sotolongo, Rex C Bentley, Rachel E Factor, William R Jeck, Laura H Rosenberger

**Affiliations:** 1 Department of Surgery, Duke University School of Medicine, Durham, USA; 2 Department of Pathology, Duke University Health System, Durham, USA; 3 Department of Surgery, Duke University Health System, Durham, USA

**Keywords:** breast cancer pathology, clinical pathology, local recurrence, phyllodes tumors, surgical margin

## Abstract

Phyllodes tumors (PTs) of the breast are rare fibroepithelial neoplasms with high rates of local recurrence (LR) for which surgical excision remains the mainstay of treatment. The recommended surgical margin width is debated and varies based on the histologic grade. We present a case of a middle-aged woman with a well-circumscribed borderline PT who underwent margin re-excision, with a distinct focus of the PT detected at a previously negative margin. Given the high rates of LR and potential for histologic upgrade at the time of recurrence, combined with the current limited understanding of peritumoral tissue and possible satellite foci, a wide negative margin remains the recommendation for borderline PTs.

## Introduction

Comprising less than 1% of all primary breast neoplasms, phyllodes tumors (PTs) are rare fibroepithelial lesions with a characteristic leaf-like appearance on histology [1]. The World Health Organization (WHO) classifies PTs as benign, borderline, or malignant based on six histopathological criteria: stromal cellularity, stromal atypia, stromal overgrowth, mitoses per 10 high-power fields (hpf), histological tumor border, and any presence of malignant heterologous elements [2]. Benign PTs are the most prevalent subtype, accounting for 70% of PT diagnoses, while borderline and malignant PTs are less common, representing 20% and 10% of cases, respectively [3,4]. While benign PTs are typically slow-growing and have histologic overlap with fibroadenomas, borderline and malignant PTs are more aggressive, with higher potential for local recurrence (LR) and distant spread [4]. Additionally, across multiple large series, 20% of benign and borderline index tumors will exhibit higher-grade histologic features and will be upgraded to a borderline or malignant classification at the time of recurrence [5]. Although less frequent, malignant PTs can be devastating, carrying a 20% risk of distant recurrence with an overall survival ranging from seven to 15 months in the metastatic setting [6,7].

Given the lack of supporting data for systemic therapy, the mainstay of treatment for PTs of all grades remains surgical excision, with consideration for radiation. In recent years, there has been a lack of consensus regarding the optimal surgical margin width for the management of PTs. Prior versions of the National Comprehensive Cancer Network (NCCN) guidelines recommend wide local excision for all PT grades, with the intention of obtaining surgical margins ≥1 cm [8]. In the current 2025 version, the NCCN guidelines state that for benign PTs, excisional biopsy or enucleation, with no intent for a negative margin, is sufficient, including in the presence of a final positive margin. Borderline and malignant PTs should still undergo wide local excision with an attempt at widely negative surgical margins [9]. However, several recent studies contend that obtaining a wide margin may not be necessary to prevent recurrence for borderline or even malignant PTs, suggesting instead that narrow negative margins are sufficient [10,11]. Since achieving a wide negative margin may require a second operation and have aesthetic implications, these findings merit careful evaluation. Nonetheless, the potential for PTs to undergo malignant transformation and the limited available data, given the rarity of the tumor, may warrant a conservative surgical approach. Here, we present a case of a patient with a borderline PT with a well-circumscribed histologic tumor border who underwent re-excision of a negative narrow medial margin (in the presence of focal positive posterior margin), revealing a distinct borderline PT satellite lesion.

## Case presentation

**Figure 1:**
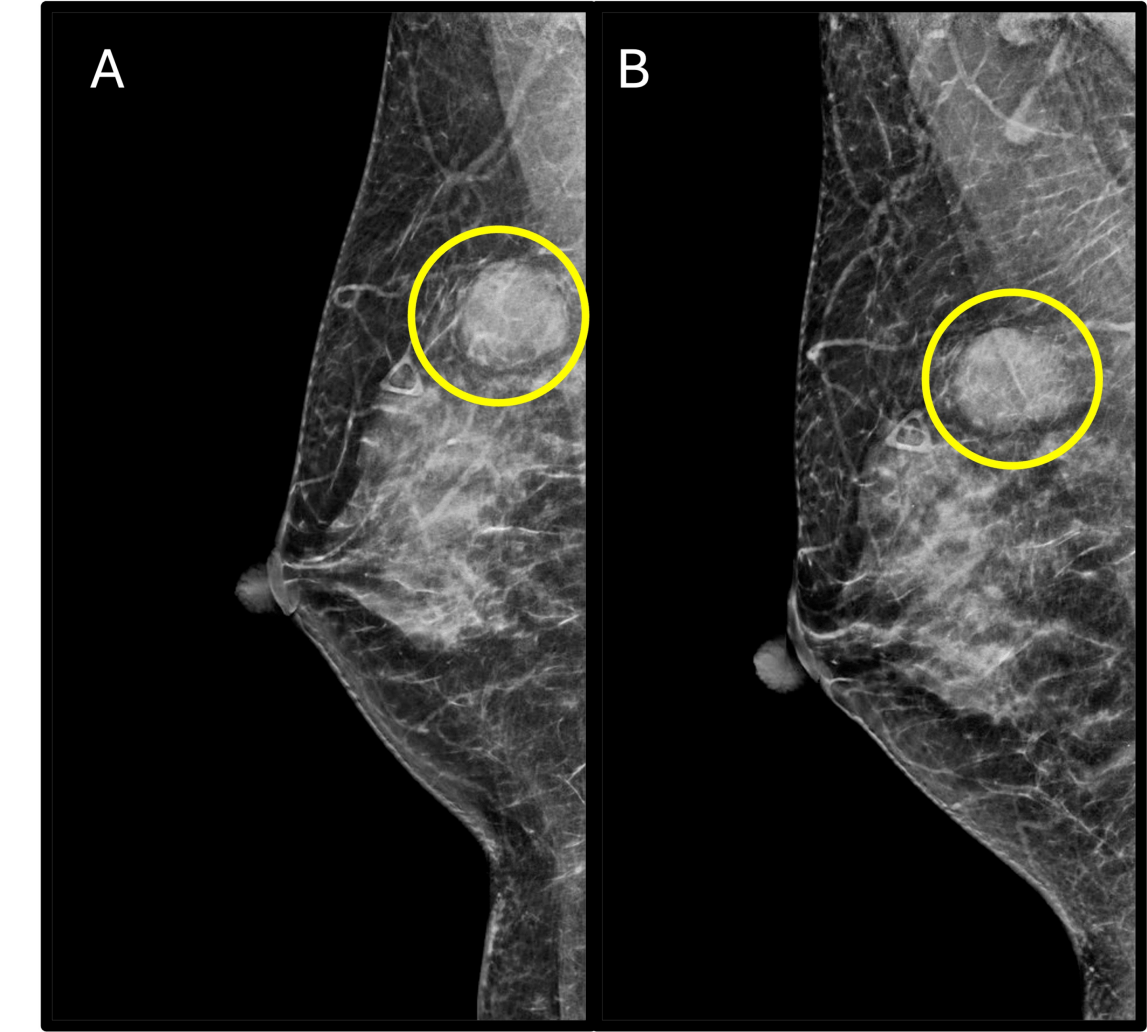
Craniocaudal (A) and mediolateral oblique (B) right breast mammograms acquired at patient presentation showing 2.4 cm circumscribed mass at 12 o’clock position, posterior depth.

A healthy woman in her 40s presented with a self-palpated mass in her right breast, initially detected two months prior. The mass did not fluctuate with the menstrual cycle and was associated with intermittent, mild pain. The patient was premenopausal, gravida 2 para 2, and had a history of breastfeeding and oral contraceptive use. Family history was significant for breast cancer in a maternal aunt and prostate cancer in her father. On clinical exam, the right breast mass was located at the 12 o’clock position, 6 cm from the nipple, and measured 2.5 cm in the largest diameter. The patient underwent a bilateral diagnostic mammogram with tomosynthesis (Figure 1) and right breast-focused ultrasound. The mammogram showed a well-circumscribed mass at the palpable site, posterior depth, not evident on a prior mammogram taken nine months earlier. There were no additional suspicious findings noted in either breast. On ultrasound, the right breast mass was parallel oriented, hypoechoic, measuring 2.4 x 1.3 x 2.2 cm with lobulated margins. A week later, the patient underwent an ultrasound-guided core needle biopsy. Pathology revealed a fibroadenoma with no evidence of atypia or carcinoma. This was deemed radiographic/pathologic concordant, and she underwent observation.

**Figure 2:**
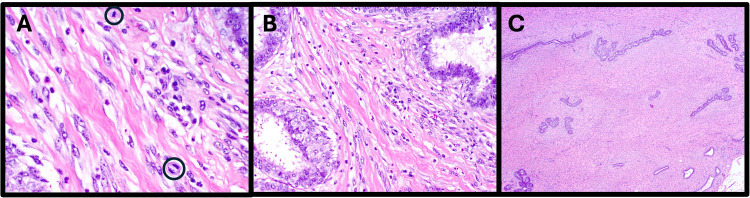
Micrographs of the borderline PT after index operation with 40x view showing 2 mitoses per 10 high-power fields (A, mitotic figures circled), 20x view showing moderately increased stromal cellularity and atypia (B), and 4x view showing no stromal overgrowth (C). PT: Phyllodes tumor

**Figure 3:**
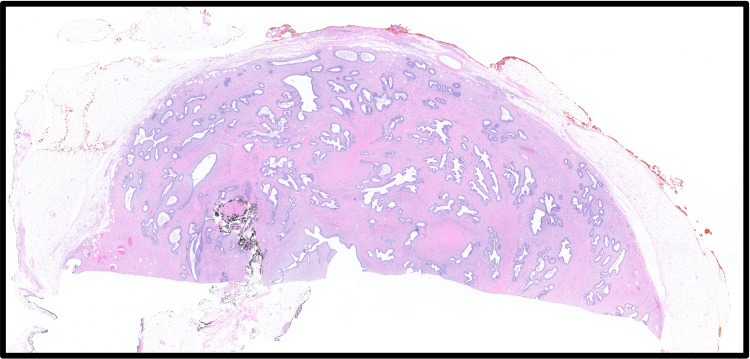
Micrograph of the borderline PT after index operation with low-power view showing a well-circumscribed histologic tumor border. PT: Phyllodes tumor

Four months later, she returned to our center with interval growth of the mass. On clinical exam, the mass measured 5.0 cm, roughly doubling in size since the time of biopsy. Given the rapid growth and overall size of the mass, there was clinical suspicion of a PT. The patient consented and underwent a right lumpectomy with intent for negative margins. Surgery was without complication. Pathology returned as a 6.0 cm borderline PT with moderate stromal cellularity, moderate stromal atypia, no stromal overgrowth, 2 mitoses per 10 hpf, a well-circumscribed histologic tumor border, and no malignant heterologous elements (Figures 2, 3). Surgical margins were positive posteriorly, negative but narrow (0.5 mm) medially, and widely negative (> 2 mm) in all other directions.

**Figure 4:**
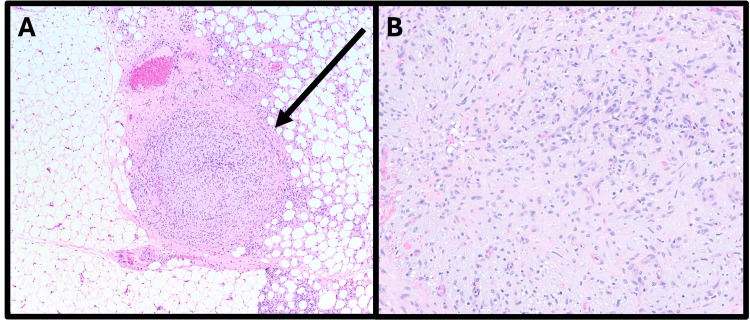
10x micrograph (A) and 20x micrograph (B) of medial margin re-excision, with the arrow pointing to the additional medial satellite foci of the tumor spatially separate from the index tumor.

The patient was discussed at a multidisciplinary sarcoma tumor board with consensus that the patient undergo re-excision of margins without adjuvant radiation therapy, given the patient’s young age and possible late effects of radiation. Given the overall size, classification as a borderline PT, and the positive margin, a margin re-excision versus mastectomy was discussed. The patient elected for a breast conserving approach with margin re-excision, and she underwent a second operation < 4 weeks from her index surgery for additional posterior and medial margin re-excisions. The prior lumpectomy cavity was easily identified as it had a small seroma present upon entry and was previously marked with surgical clips at the time of the index operation. On final pathology, the previously positive posterior margin was negative; however, the previously negative but close medial margin had a 1 mm residual foci of the PT that was discontinuous with the index tumor (Figure 4).

Germline genetic testing was performed with a custom common hereditary cancer panel and sarcoma panel (62 genes), which resulted as negative (variant of unknown significance (VUS) in *WRN*, c.1760A>C (p.Tyr587Ser), heterozygous). At present, the patient is three months into surveillance with no evidence of disease. She will undergo heightened surveillance with an annual tomosynthesis mammogram and a focal ultrasound of the lumpectomy site every six months for two years. After that, she will continue annual tomosynthesis mammogram and ultrasound through five years.

## Discussion

PTs of the breast are uncommon fibroepithelial neoplasms that carry a significant risk of LR, with surgery being the cornerstone of treatment. Optimal surgical margin status to minimize LR rates has been a subject of debate in recent years. In past versions of the NCCN guidelines, wide local excision with > 1 cm margins was recommended for all PT grades, including benign PTs. However, in the past decade, multiple large cohort studies have challenged the recommendation for such margins. When pooling and analyzing all PT subtypes together, multiple studies such as Yom et al. (N = 285) and Rosenberger et al. (N = 550) have found no association between margin status and LR rate, a result likely found based on the high distribution of benign PTs encompassing these cohorts (67-69%) and low event rates in their series (3.3%, 7.0%) [4,12]. Moo et al. (N = 246) and Kim et al. (N=193) discovered the same trend when assessing a cohort of benign PTs only, also with only rare recurrence events reported (1.9%, 3.4%) [13,14]. In addition, a 2019 meta-analysis found that a positive margin was not associated with LR risk in benign PTs [5]. Reflecting these collective findings, the NCCN guidelines were updated, stating that excisional biopsy with permissive positive margins is sufficient for benign PTs, while wide local excision with an attempt at margins > 1 cm remains indicated for borderline and malignant subtypes [9].

While multiple studies find no association between a positive margin and LR for benign PTs, potentially due to short follow-up and low recurrence rates, a number of large, granular series still do. In a review performed by Spitaleri et al. on 5,530 PT cases, positive margin status was found to be a significant predictor of benign PT recurrence [15]. Similarly, a recent population-based national cohort of 1,908 benign PTs identified by a unified nationwide pathology database found a doubling of LR rate with positive surgical margins as compared to negative (8.9% vs. 4.0%) [16]. While these authors acknowledge the significant and clinically meaningful increase in LR with positive margins, they conclude that the overall LR rate is low, even in the presence of positive margins, obviating the need for margin re-excision.

Recent studies continue to challenge these updated recommendations as too conservative. While mostly acknowledging the importance of a negative margin for borderline and malignant PTs, some authors argue that a wide negative margin may not be necessary for preventing recurrence [10,11]. In a study analyzing 165 PT cases, Changchit et al. found that while margins < 1 mm increased the LR rate of borderline PTs, LR rates did not differ between patients with narrow negative margins (1 mm to 9 mm) and wide negative margins (> 1 cm) [10]. Similarly, in a cohort of 921 patients, Bartels et al. report similar LR rates between patients with narrow (0 to 1 mm) and > 1 mm margins, concluding that re-excision of narrow margins may not be necessary for borderline or malignant PTs [11]. Additional studies done by Yoon et al. and Genco et al. demonstrate similar results [17,18]. As is the challenge with research involving rare tumors, these series are severely limited by being retrospective, having limited cohort sizes, having few events, pooling of PT subtypes in the analyses, and an inability to perform multivariate analyses without overfitting models. Nonetheless, these studies, even with their limitations, raise the question: should narrow margins be accepted for all subtypes of PTs?

Classically, PTs present as single well-circumscribed lesions both macroscopically and microscopically. Cases of multifocal disease are exceedingly scarce in the literature. However, given the rarity of this tumor and only the recent adoption of standard histopathologic reporting protocol in 2022, current understanding of histologic tumor borders, peritumoral stroma, and presence of satellite tumor foci remains limited [19]. In the case we present herein, a satellite focus of tumor was found on the edge of a re-excised medial margin specimen that was deemed narrowly negative at index surgery, raising the suspicion that accepting narrow negative margins may miss areas of satellite disease. PTs, in contradistinction to breast adenocarcinoma, are rarely treated with adjuvant whole breast radiation. Therefore, satellite nodules are likely to lead to a local recurrence in a short interval.

In a single case report from 1978, Salm describes a multifocal PT with isolated tumor nodules located < 1 cm from the primary tumor, hypothesizing this as a mechanism by which the PT achieves locoregional spread [20]. Thirty years later, Slodkowska et al. analyzed a cohort of 178 patients diagnosed with fibroepithelial lesions (75% characterized as PTs) and found that 60 tumors had satellite or bulging nodules, with satellite nodules more frequently identified in the PT subgroup [21]. Additionally, tumors with pushing borders without nodules had a significantly improved recurrence free survival than tumors with infiltrative borders with nodules, and the presence of satellite nodules significantly predicted positive margin status [21].

When considered in the context of the existing body of literature, this case raises several important questions. How often are PT satellite foci present? When present, do foci typically occur < 1 cm from the index lesion so they can be captured on wide margin excision? Clinically, does the presence of satellite foci portend recurrence or correlate with more aggressive tumor biology within the satellite foci? Until more information is elucidated, we caution against readily accepted narrow negative margins for borderline or malignant PTs and encourage increased awareness of possible peritumoral disease foci.

## Conclusions

In summary, our case and a review of the literature caution against narrow negative margins for borderline or malignant PTs. Studies that group all subtypes together to analyze LR likely overrepresent benign tumors that are less aggressive at baseline. While frequency is unknown, opting for narrow negative margins could leave behind satellite lesions, potentially subjecting a patient to increased risk of LR or the risk of malignant transformation.
